# Surgical and nonsurgical treatment of hip and knee osteoarthritis: a 2026 perspective

**DOI:** 10.2340/17453674.2026.45570

**Published:** 2026-03-03

**Authors:** Jens LAIGAARD, Tazio MALEITZKE, Søren T SKOU, Inger MECHLENBURG, Thomas BANDHOLM, Lise C BERG, Søren OVERGAARD

**Affiliations:** 1 Department of Orthopaedic Surgery and Traumatology, Bispebjerg University Hospital, Copenhagen, Denmark; 2 Department of Clinical Medicine, University of Copenhagen, Copenhagen, Denmark; 3 Trauma Orthopaedic Research Copenhagen Hvidovre (TORCH), Department of Orthopaedic Surgery, Copenhagen University Hospital-Amager and Hvidovre, Hvidovre, Denmark; 4 Charité – Universitätsmedizin Berlin, Corporate Member of Freie Universität Berlin and Humboldt-Universität zu Berlin, Center for Musculoskeletal Surgery, Berlin, Germany; 5 Berlin Institute of Health at Charité – Universitätsmedizin Berlin, Julius Wolff Institute, Berlin, Germany; 6 Research and Implementation Unit PROgrez, Central and West Zealand Hospital, Slagelse, Denmark; 7 Center for Muscle and Joint Health, University of Southern Denmark, Odense, Denmark; 8 Department of Orthopaedic Surgery, Aarhus University Hospital, Aarhus, Denmark; 9 Department of Public Health, Section of Sport Science, Aarhus University, Aarhus, Denmark; 10 Department of Clinical Medicine, Aarhus University, Aarhus, Denmark; 11 Physical Medicine & Rehabilitation Research – Copenhagen (PMR-C), Department of Physical and Occupational Therapy, Copenhagen University Hospital Amager-Hvidovre, Hvidovre, Denmark; 12 Clinical Orthopedic Research Hvidovre (CORH), Department of Orthopedic Surgery, Copenhagen University Hospital Amager-Hvidovre, Hvidovre, Denmark; 13 Department of Veterinary Clinical Sciences, Faculty of Health and Medical Sciences, University of Copenhagen, Taastrup, Denmark

This *Perspective* is based on the symposium entitled “Treatment of Osteoarthritis,” held at the 2025 annual Snekkersten Research Retreat, organized by the Graduate PhD Programme Basic and Clinical Research in Musculoskeletal Sciences (MUSKOS) at the University of Copenhagen, in collaboration with the Research in OsteoArthritis Denmark Clinical Academic Group (ROAD CAG). The symposium was centered on the clinically important question: Surgical or nonsurgical treatment of hip and knee osteoarthritis (OA)?

Hip and knee OA are highly prevalent and characterized by significant pain, disability, and reduced quality of life, thus representing a major burden for healthcare systems worldwide. People with hip and knee OA present with considerable variation in symptoms and disease progression. Surgeons are often surprised that only a minority of patients diagnosed with OA will end up undergoing joint replacement surgery, probably because they typically see patients at advanced disease stages with severe symptoms [1]. Treatment begins with nonsurgical interventions such as patient education, exercise, weight management, and analgesics, which remain the primary therapies for most, although surgery may be indicated in some [1,2]. Joint replacements are effective, but also irreversible and associated with serious adverse events [3,4]. Effective nonsurgical treatments are therefore in high demand.

We present perspectives on state-of-the-art practice and future perspectives of surgical and nonsurgical treatment of hip and knee OA.

## Hip OA

In hip OA, meta-analyses have confirmed that exercise improves pain and function, although the effects were small and may be explained by the placebo effect, as blinding is inherently difficult in trials investigating physical exercise [5-7]. The most recent network meta-analysis found very sparse evidence to suggest that 1 exercise modality is superior in terms of pain, function, physical performance measures, and quality of life [8]. Several recent randomized controlled trials (RCTs) have also compared the effect of different exercise modalities for patients with hip OA, but none have shown clinically meaningful superiority for their primary endpoints.

In patients with severe hip OA, hip replacement resulted in clinically important improvements in pain and hip function at 6 months compared with progressive resistance training [3]. Hip replacement did not lead to clinically important greater improvement in activity level, gait speed, or sit-to-stand function at 6 months. However, 77% of the patients randomized to resistance training had undergone hip replacement within 2 years of randomization [3].

## Knee OA

In knee OA, exercise and weight management are established as beneficial for pain, physical function, and quality of life [7-9]. However, the effect estimates are based on trials without placebo groups and are therefore difficult to distinguish from contextual effects [6]. A recent network meta-analysis comparing the effect of various exercise modalities on pain, function, gait performance, and quality of life found no clear superiority of a single modality [10]. This conclusion likely resulted from the large number of primary endpoints, each analyzed with a limited number of participants. Aerobic exercises, for example, contributed to the analyses with only between 16 and 280 participants from 34 different RCTs [10].

In obese patient with moderate-to-severe pain from knee OA, treatment with a glucagon-like peptide-1 (GLP-1) receptor agonist improves pain, although evidence is lacking in patients with an indication for knee replacement [9].

In severe knee OA, total knee replacement followed by nonsurgical care gave significantly larger improvements in pain, function, and quality of life at 3 months, compared with nonsurgical care alone [4]. However, both groups improved, and patients randomized to nonsurgical care alone experienced significantly fewer serious adverse events. Moreover, only one-third had undergone surgery within 2 years of randomization. Similarly, a more recent exercise RCT found that only 43% of patients underwent surgery within 2 years, despite having an indication for knee replacement at baseline [11].

## Future of OA treatment

To date, no approved therapy can reverse or halt OA progression. Pre-clinical studies have identified various disease-modifying OA drugs (DMOADs) that effectively prevent extracellular matrix and cartilage degradation in vitro and in vivo. Yet the subsequent phase III trials investigating these DMOADs have mostly failed to meet their primary endpoints. However, recent exploratory analyses of these trials have suggested improvements in pain in certain subpopulations, such as specific OA endotypes [12]. If these results can be reproduced in confirmatory RCTs, more personalized therapies, based on OA pheno- and endotypes, may play a role in future practice.

Orthobiologics are biological substances mostly used as autologous intra-articular therapies. Mesenchymal stromal cells have been tested extensively, yet clear clinical benefits are debated. While a recently performed RCT showed no differences in clinical improvements between mesenchymal stromal cells (from different sources) and corticosteroid injections [13], a post-hoc analysis proved to offer greater therapy responsiveness following autologous compared with allogenic and corticosteroid therapies [12]. Platelet-rich plasma, an autologous blood product high in growth factors, is widely used in clinical practice to potentially control pain and intraarticular inflammation in OA patients. Data from large RCTs show no effect beyond placebo [14,15]. Systematic reviews and meta-analyses confirm conflicting evidence on efficacy of platelet-rich plasma and point to a lack of standardization of products.

Genicular artery embolization is a minimally invasive and catheter-based intervention in knee OA where pathological periarticular vessels are temporarily or permanently embolized. Large prospective case series have shown improvements in pain, function, and quality of life up to 12 months post-treatment [16]. Previous RCTs investigating genicular artery embolization had small sample sizes and inconclusive results, but 2 large-scale RCTs are currently underway (NCT05423587, NCT05476913).

Knee joint distraction with a dedicated joint-spanning external fixator has been applied to knee OA patients in the Netherlands for many years. Observational studies have shown clinically relevant and sustainable improvements in cartilage volume and pain scores. While smaller prospective studies have shown preliminary safety and efficacy, high-quality evidence is still missing. A large British RCT was stopped early due to difficulties with patient recruitment and communication, but the Dutch GODIVA trial (NCT06113549) is currently randomizing 1,200 patients to either knee joint distraction or total knee replacement [17].

## Discussion

Evidence suggests that nonsurgical treatment is relevant, even in patients considered eligible for joint replacement. The effects of physical exercise on OA symptoms are difficult to isolate from contextual effects such as the social benefits of group sessions or reassuring discussions with a physiotherapist. Yet exercise undeniably improves general health and may allow symptoms to regress without surgery. Thus, the clinical challenge lies mainly in balancing nonsurgical strategies with timely surgery. Coordinated care pathways that connect primary care, physiotherapy, and orthopedic surgery offer the most effective way to ensure that each patient receives the right treatment at the right time. For orthopedic surgeons, this means that nonsurgical treatment should increasingly be used in surgical decision-making, particularly in young patients and patients at high risk of adverse events following surgery [3,4,11].

For patients with both hip and knee OA, no single exercise modality outperforms others in a clinically meaningful way. The implication is clear: exercise programs should be tailored to each patient’s preference and needs, with emphasis on adherence. When non-surgical interventions fail, joint replacement can substantially improve pain and function. The term “end-stage” OA is often used to describe patients where joint replacement is inevitable. However, the term is ill-defined and many patients described as having end-stage OA could probably postpone surgery for a substantial amount of time [2-4,11]. Postponement of surgery may, however, not always be desirable and should be seen in a broader perspective, taking the patient’s symptoms, ability to work, physical capabilities, quality of life, and societal costs into account. Development of improved diagnostic tools for OA classification, with clinical, radiographic, and biochemical measures, is warranted to improve interpretation of trial results and optimize the timing of surgery [2].

Results from large-scale RCTs are yet to confirm the effects of knee joint distraction, genicular artery embolization, orthobiologics, and DMOADs. Joint efforts are required to implement and rethink current and future OA care pathways.

## Notes

TM, STS, and IM presented at the symposium. LCB and SO chaired the symposium. TB is the head of the MUSKOS graduate PhD program who arranged the symposium. JL, TM, STS, IM, and SO drafted the manuscript, which was critically revised by all authors.

The authors would like to thank Lea Parmark for planning and facilitating the 2025 MUSKOS Snekkersten Meeting.

Handling co-editor: Li Felländer-Tsai

*Acta* thanks an anonymous reviewer for help with peer review of this manuscript.
